# Prescription Department

**Published:** 1892-11

**Authors:** 


ASTHMA.—*Dr. N. L. Clark.*
R. Potass. iodid., dr. ijss.
Ext belladonnæ fl., dr. iss.
Ext. lobeliæ, fl. dr. iij.
Ext. grindel. rob. fl., oz. i.
Syr. tolutani, ad oz. iv.
M. Sig. Dr. i. every hour when necessary.

DIPHTHERIA AND CROUP.—*Dr. Ellis.*
R. Potass. chlorat., scruple ij.
Tr. ferri. chlor., m xl.
Syr. simplicis, oz. ss.
Aquæ, ad oz. ij.
M. Sig Two teaspoonfuls every three or four hours.

SPASMODIC CROUP.—*Dr. Ellis.*
R. Pot. bromid.
Chloral hydrat., aa scruple ij.
Syrup acaciæ, oz. ij.
M. Ft. Sol. Sig. Dr. i. or less, according to age.

DYSENTERY OF CHILDREN.—*Dr. Ellis.*

R. Ol. ricini, dr. i.

Pulv. acaciæ, scruple i.

Syrupi, dr. i.

Tr. opii, m iv.

Aquæ flor. aurant., dr. vj.

NIGHT SWEATS OF PHTHISIS.—*Dr. Barlow.*
R. Zinci sulph. gr. vj.
Ext hyoscyami, gr. xxiv.
Divide in pil. No. vj.
Sig. One at bed-time.

CHRONIC PNEUMONIA.—*Prof. Da Costa.*
R. Liq. pot. arsenitis, dr. ss.
Ammon. iodid., dr. ij.
Tr. cinchonæ co., oz. iij.
M. Sig. Dr. i. three times a day. Small blisters ought to be frequently applied along with the above.

CONSUMPTIVE COUGH.—*Dr. Morton.*
R. Ac. hydrocyanic dil. m xvi.
Mucil. acaciæ, oz. i.
Syr. tolutani, oz. ss.
Aq. ad., oz. viij.
M. Sig. Tablespoonful every three hours.

IN TYPHOID FEVER.—*Prof. Bartholow.*
R. Tr. iodini, dr. ij.
Ac. carbol., dr. i.
M. Sig. One to three drops three times a day.

Diarrhœa of Typhoid Fever.—*Prof. Bartholow.*

℞. Argent. nit., gr. iij.
Pulv. opii.
Pulv. ipecac, aa. gr. vj.
M. Divide in pil No. xij.
Sig. One every four to six hours.

Sedative and Stimulant.—*Dr. Tilt.*

℞. Chloroform, oz. ss.
Spts. turpentine, oz. i.
Camphor, oz. ij.
Oil lavand., m. xx.
Olive oil ad., oz. iv.
M. Ft. Liniment.

Metrorrhagia.—By *Dr. Taylor*, Philadelphia, Pa.

℞. Ergotinæ, gr. xl.
Ext. nucis vomicæ, gr. x-xx.
Ferri redacti, gr. xl.
M. Et. ft. pil. No. xx. S. One pill three times a day.

Dysmenorrhœa.—*Dr. Greenhalgh.*

℞. Ac. phosphor. dil., dr. i.
Liq. strychniæ, m. xv.
Spirits chloroformi, dr. ij.
Syrup aurantiæ, dr. iv.
Aq ad., oz. vi.
M. Sig. Oz. i. three times a day.

Intestinal Hemorrhage in Typhoid Fever.—*Dr. Murchison.*

℞. Ac. tannic, gr. x.
Tr. opii., m. x.
Spirits terebinth., m. xv.
Mucil. acaciæ, dr. ij.
Tr. chloroformi, m. xx.
Ac. minth. pip. ad., oz. i.
M. Ft. Haustus. Sig. To be taken at bed-time.

Rheumatism and Neuralgia.—*Dr. Wyman.*

℞. Tr. Aconiti, dr. i.
Tr. Opii,
Chloroformi, aa. oz. ss.
Lin. saponis, ad. oz. vi.
M. Ft. Liniment.

Indolent Bowels in Aged Persons.—*Dr. Robinson.*

℞. Pulv. scammon., dr. ss.
Ext. aloes, dr. i.
Bals. peru, gr. x.
Ol. carui, m. x.
M. Ft. in pil. No. xx. Sig. One at night.

